# The Secreted Virulence Factor NADase of Group A *Streptococcus* Inhibits P2X7 Receptor-Mediated Release of IL-1β

**DOI:** 10.3389/fimmu.2019.01385

**Published:** 2019-06-18

**Authors:** Elsa Westerlund, Christine Valfridsson, Daisy X. Yi, Jenny J. Persson

**Affiliations:** ^1^Immunology Section, Department of Experimental Medical Sciences, Lund University, Lund, Sweden; ^2^Division of Immunology and Pathogenesis, Department of Molecular and Cell Biology, University of California, Berkeley, Berkeley, CA, United States

**Keywords:** Group A *Streptococcus*, NADase, P2X7, IL-1β, membrane permeabilization

## Abstract

The common human pathogen Group A *Streptococcus* (GAS) causes superficial as well as invasive, life-threatening diseases. An increase in the occurrence of invasive GAS infection by strains of the M1 and M89 serotypes has been correlated with increased expression of the genetically and functionally linked virulence factors streptolysin O (SLO) and β-NAD^+^-glycohydrolase (NADase). NADase affects host cells differently depending on its location: its SLO-dependent translocation into the cytosol can lead to cell death through β-NAD^+^ depletion, while extracellularly located NADase inhibits IL-1β release downstream of Nlrp3 inflammasome activation. In this study, we use a macrophage infection model to investigate the NADase-dependent inhibition of IL-1β release. We show that bacteria expressing a functional NADase evade P2X7 activation, while infection with a NADase-deficient GAS strain leads to a P2X7-mediated increase in IL-1β. Further, our data indicate that in the absence of NADase, IL-1β is released through both P2X7-dependent and -independent pathways, although the precise mechanisms of how this occur are still unclear. This study adds information about the mechanism by which NADase regulates inflammasome-dependent IL-1β release, which may in part explain why increased NADase expression correlates with bacterial virulence.

## Introduction

The human pathogen Group A *Streptococcus* (GAS; *Streptococcus pyogenes*) causes diseases ranging in severity from mild pharyngitis and impetigo to life-threatening streptococcal toxic shock syndrome and necrotizing fasciitis (NF), and is estimated to cause about 700 million superficial and 650,000 invasive infections yearly (1). Here, we investigate how this pathogen modifies the release of the pro-inflammatory cytokine interleukin (IL)-1β, and which role the purinergic receptor P2X7 plays in this regulation.

The pore-forming protein streptolysin O (SLO) and its co-toxin, the enzyme β-NAD^+^-glycohydrolase (NADase, also called SPN), are two secreted proteins that are part of an impressive arsenal of virulence factors employed by GAS (1, 2). SLO inserts into host cell membranes, creating large pores that may result in cell death (3) or activation of the Nlrp3 inflammasome (4, 5). In addition, SLO can confer translocation of NADase into the cell cytosol (6), where this NAD^+^-hydrolyzing enzyme may deplete cellular energy stores leading to cell death (7). SLO and NADase have been assigned a number of functionally linked roles in streptococcal pathogenesis (8–11), and their importance was further highlighted as their increased expression in specific clones of the M1 and M89 serotypes was linked to a rapid surge in invasive disease and the global dissemination of these strains (12).

As part of our first line of defense against pathogens, innate immune cells utilize pattern-recognition receptors (PRRs) to recognize conserved bacterial and viral structures, or molecules that signal cellular distress (13). This can lead to production of proinflammatory cytokines such as IL-1β, a potent mediator of inflammation and a factor involved in the host protection response (14). IL-1β is synthesized as an inactive pro-form (pro-IL-1β) that can be cleaved to generate active IL-1β, e.g., by the cysteine protease caspase-1 within the so-called inflammasomes (15). An inflammasome commonly contains caspase-1, the adaptor molecule ASC and a sensor protein such as Nlrp3. In murine macrophages, activation of the Nlrp3 inflammasome requires two sequential signals. Signal one (“priming”) mediates production of the Nlrp3 and pro-IL-1β proteins and induces essential post-translational protein modifications, often after PRR sensing of microbial ligands or endogenous danger signals. *In vivo*, priming likely occurs through multiple receptors; *in vitro* however signal one is most commonly mediated by LPS through TLR4 to allow isolated studies of signal two. Signal two (“activation”) leads to assembly of the inflammasome complex, activation of caspase-1 and subsequent cleavage and release of IL-1β (16). Although it has been shown that many triggers of the Nlrp3 inflammasome, including SLO, induce efflux of cytosolic K^+^ (17), the exact mechanisms leading to Nlrp3 activation are not known.

Unlike most secreted proteins, IL-1β lacks a conventional N-terminal signal peptide and is instead secreted through unconventional release mechanisms. There are currently a number of suggested pathways for IL-1β release, roughly divided into vesicular and non-vesicular routes (18), some of which have been linked to the P2X7 receptor (19). Notably, little is known about the IL-1β release pathways involved in situations where several stimuli may be present, such as in response to bacterial infections.

The role of IL-1β in GAS infection is complex: on one hand the IL-1 receptor (IL-1R) antagonist Anakinra increases the risk of acquiring NF (20), indicating a protective role for IL-1β in this syndrome. On the other hand, tissue damage and hyperinflammation due to uncontrolled IL-1β levels illustrates its detrimental effects and indicates that both host and pathogen benefit from a fine-tuned response (21). In a recent report we describe a novel function for NADase present in the extracellular compartment: inhibition of IL-1β release downstream of SLO-mediated inflammasome activation. Using a wild type (wt) GAS strain originating from the globally dispersed M1 clone, and an isogenic mutant strain expressing enzymatically inactive NADase (*nga*(G330D)), we could show that NADase inhibits the release of Nlrp3 inflammasome-dependent mature IL-1β (5). This novel role for NADase could be assigned to the extracellularly located fraction of the toxin and represents the first description of a function for non-translocated NADase. Curiously, NADase-dependent suppression of IL-1β could not be explained by differential transcriptional or translational responses or alterations in activation of the inflammasome as such, as intracellular pro-IL-1β levels and caspase-1 activation were similar in macrophages infected with wt or *nga*(G330D) streptococci (5). Here we explore this phenomenon further, and although an exact mechanism is still lacking, our results indicate that the expression of a functional NADase toxin permits the bacteria to evade activation of the P2X7 receptor, which explains the observed decrease in IL-1β secretion. Indeed, a NADase deficient streptococcal strain activates P2X7 in infected macrophages, leading to membrane permeabilization and increased release of IL-1β.

## Results

### A Group A *Streptococcus* Strain Lacking NADase Activity Induces a P2X7-Dependent IL-1β Release Pathway

**Figure 1:**
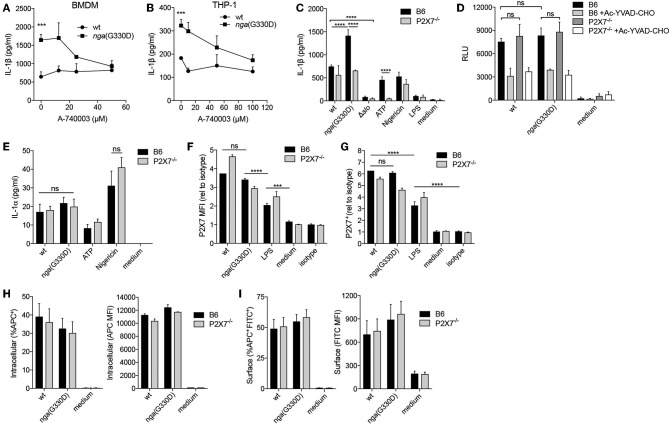
A Group A *Streptococcus* strain lacking NADase activity induces a P2X7-dependent IL-1β release pathway. **(A)** LPS-primed B6 BMDMs or **(B)** differentiated THP-1 cells were infected with wt or *nga*(G330D) GAS in the presence of A-740003. **(C)** BMDMs of the indicated genotypes were LPS-primed and infected with wt, *nga*(G330D) or Δ*slo* GAS, or left uninfected as indicated (LPS). **(D,E)** BMDMs of the indicated genotypes were LPS-primed and infected with wt or *nga*(G330D) GAS, or left untreated (medium). Supernatants were analyzed for **(A–C)** IL-1β levels, **(D)** caspase-1 activity or **(E)** IL-1α levels by ELISA, a luminescence-based assay or cytometric bead array (CBA), respectively. **(F,G)** BMDMs of the indicated genotypes were LPS-primed and infected with wt or *nga*(G330D) GAS, only LPS-primed, or left untreated and analyzed for **(F)** P2X7 receptor surface levels or **(G)** frequency of P2X7 expressing BMDMs by flow cytometry (FACS). **(H,I)** LPS-primed BMDMs of the indicated genotypes were infected with wt or *nga*(G330D) GAS stained with Alexa Fluor 600nm and analyzed for **(H)** intracellular or **(I)** surface-associated bacteria by FACS. ATP and Nigericin were used as controls for P2X7 knockout and Nlrp3 inflammasome activation, respectively. Graphs show means plus SD for **(A–E)** triplicate or **(F–I)** duplicate technical replicates and are representative of at least three independent experiments. **(F,G)** Values are normalized to isotype control. LPS, LPS-primed but uninfected. Significant differences are indicated by asterisks as follows: ^***^P < 0.001; ^****^P < 0.0001. Values that are not significantly different are indicated (ns).

The P2X7 receptor has been implicated in the regulation of different secretory pathways governing the unconventional release of IL-1β (19). To analyze the potential involvement of P2X7 in IL-1β release during GAS infection, we infected murine bone marrow derived macrophages (BMDMs) with wt or *nga*(G330D) bacteria in the presence of the P2X7-specific antagonist A-740003 (22). Of note, in all infection experiments we use LPS to prime the BMDMs for inflammasome activation. While P2X7 inhibition did not affect IL-1β release from BMDMs infected with wt bacteria, the IL-1β released upon *nga*(G330D)-infection was dose-dependently reduced, plateauing at levels similar to those induced by wt bacteria (Figure 1A). Correspondingly, P2X7 inhibition during infections of the human monocytic cell line THP-1, differentiated into adherent macrophages, exhibited similar IL-1β release patterns as BMDMs (Figure 1B).

To further corroborate the involvement of P2X7, we performed wt and *nga*(G330D) infections in BMDMs from P2X7^−/−^ mice that present a non-functional P2X7 protein on their surface (23). In agreement with the experiments using the P2X7 inhibitor, B6 and P2X7^−/−^ BMDMs released comparable levels of IL-1β after infection with the wt strain, while *nga*(G330D) bacteria induced increased IL-1β secretion from B6 but not from P2X7^−/−^ BMDMs. As expected, LPS-priming alone did not support release of IL-1β (Figure 1C).

No IL-1β was released from either B6 or P2X7^−/−^ cells when infected with bacteria lacking SLO expression, suggesting that inflammasome activation proceeds along the same pathway in cells of both genotypes. In line with our previously reported data (5), we detected no difference in total caspase-1 activity when comparing cells infected with wt or *nga*(G330D) bacteria (Figure 1D). Similarly we measured comparable levels of caspase-1 activity in infected B6 and P2X7^−/−^ macrophages (Figure 1D), suggesting that the observed alterations in released IL-1β cannot be explained by differences in inflammasome activation *per se*. Importantly, P2X7^−/−^ BMDMs reacted normally to the K^+^ ionophore Nigericin (Figure 1C), demonstrating that these cells respond to stimuli not sensed by the P2X7 receptor. In addition, B6 and P2X7^−/−^ macrophages released similar levels of IL-1α upon wt and *nga*(G330D) GAS infection (Figure 1E), suggesting that P2X7-deficiency does not affect the priming of BMDMs.

In macrophages, P2X7 receptors continuously traffic between different cellular compartments and may exhibit surface upregulation upon e.g., infection (24). We hypothesized that streptococcal NADase might affect these trafficking pathways and thus inhibit P2X7-dependent IL-1β release by decreasing the presence of the receptor at the cell surface. While P2X7 was upregulated by LPS priming and further increased after bacterial infection (Figure 1F) wt- and *nga*(G330D)-infected macrophages increased surface expression of P2X7 to a similar extent (Figures 1F,G). Thus, P2X7 trafficking to the cell surface was similarly affected by wt and *nga*(G330D) GAS.

The P2X7 receptor has also been ascribed scavenger properties mediating uptake of bacteria, a function that is disrupted in the P2X7^−/−^ mice used in this study (25). To investigate whether B6 and P2X7^−/−^ BMDMs differ in ability to internalize GAS, we infected BMDMs with bacteria labeled with a fluorescent ester (Alexa Fluor 660) and subsequently stained the cells using a FITC-conjugated anti-streptococcus antibody. Using flow cytometry, we could identify cells harboring intracellular (single positive for Alexa Fluor) and/or cell-associated (double positive for Alexa Fluor and FITC) bacteria. However, we found no alterations in bacterial uptake or the numbers of surface associated bacteria between B6 and P2X7^−/−^ BMDMs, nor between wt- and *nga*(G330D)-infected cells (Figures 1H,I). Thus, the P2X7-dependent differences we observe in IL-1β release are unlikely to be explained by the scavenging properties of this receptor. Taken together, this data suggest that GAS induce SLO-mediated inflammasome-dependent IL-1β release independently of the P2X7 receptor, but that bacteria expressing an enzymatically dead NADase trigger an additional and P2X7-dependent pathway leading to increased secretion of IL-1β. Moreover, the role of P2X7 in driving this additional release does not relate to differences in surface expression of the receptor or its ability to internalize bacteria.

### P2X7-Dependent IL-1β Release Occurs Independently of Extracellular ATP or β-NAD^+^ Cleavage Products

**Figure 2:**
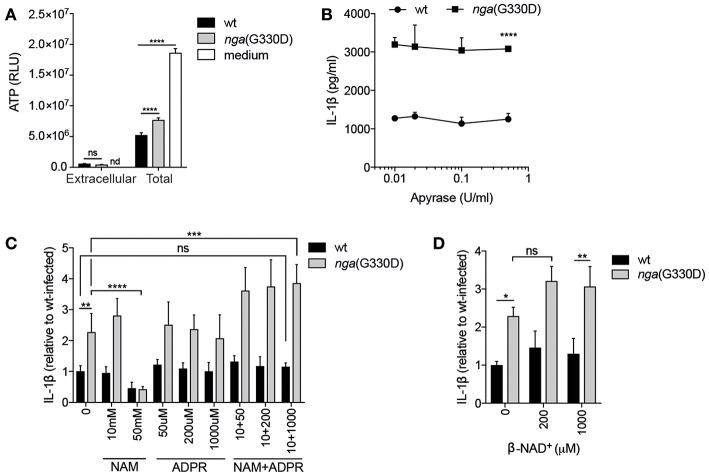
P2X7-dependent IL-1β release occurs independently of extracellular ATP or β-NAD^+^ cleavage products. **(A)** B6 BMDMs were LPS-primed and infected with wt or *nga*(G330D) GAS, or left untreated (medium), and ATP content analyzed by a luminescence-based assay. **(B–D)** LPS-primed B6 BMDMs were infected with wt or *nga*(G330D) GAS in the presence of **(B)** apyrase, **(C)** nicotinamide (NAM), ADP-ribose (ADPR), NAM+ADPR or **(D)** β-NAD^+^. **(C,D)** Values are normalized to wt-infected BMDMs. Supernatants were analyzed for IL-1β by ELISA. Graphs show means plus SD for triplicate technical replicates and are representative of at least three independent experiments. Significant differences are indicated as follows: ^*^P < 0.05; ^**^P < 0.01; ^***^P < 0.001; ^****^P < 0.0001. Values that are not significantly different are indicated (ns). Values that were below detection limit are indicated (nd).

It has been reported that ATP may be released from macrophages through open pores generated after activation of P2X7 (26), creating an autocrine ATP-P2X7-activation loop. We hypothesized that our two bacterial strains might induce differential, P2X7-dependent or -independent, release of ATP, which could lead to differences in secretion of IL-1β. However, we observed similar and very low levels of extracellular ATP during infection with either bacterial strain, even though total ATP levels were easily detectable in infected cells (Figure 2A). Untreated cells contained yet higher levels of ATP, agreeing with previous studies showing decreased intracellular ATP levels in response to infection (27, 28). Also in line with previous data (11), we observed a significant difference in total ATP levels in wt compared to *nga*(G330D) infected cells (Figure 2A). However, this difference between the two bacterial strains remained in P2X7^−/−^ cells (Supplementary Figure S1A) also disqualifying altered internal ATP levels as an explanation for differential IL-1β release. In addition, the use of the ATP-hydrolyzing enzyme Apyrase had no effect on IL-1β levels during GAS infection, although the enzyme readily hydrolyzed ATP (Figure 2B and Supplementary Figure S1B).

When streptococcal NADase hydrolyses β-NAD^+^, it generates nicotinamide (NAM) and ADP-ribose (ADPR), both substances with documented effects on eukaryotic cells (29, 30). If a NADase cleavage product mediates inhibition of P2X7-dependent IL-1β release, then the addition of them during *nga*(G330D) infection (where they should not be generated by the bacteria) should lead to a decrease in IL-1β. However, we did not observe such a decrease upon addition of either substance on its own, or in combination of the two (Figure 2C). High concentrations of NAM did inhibit IL-1β release upon infection with both wt and *nga*(G330D) bacteria. However, as it also affected the secretion of non-inflammasome cytokines like IL-6 (Supplementary Figure S1C), we believe that this inhibition is the result of a more general effect and should not be interpreted as NAM acting as an inhibitor of P2X7. Relatedly, we hypothesized that β-NAD^+^, which is not hydrolyzed during infection with *nga*(G330D) bacteria, might drive the P2X7-dependent increase in IL-1β release. We expected that, if true, the addition of this compound during *nga*(G330D) infections would increase IL-1β secretion even further. However, supplementing infections with β-NAD^+^ had no significant effect on IL-1β release (Figure 2D). Taken together, our data suggest that during infection with NADase-deficient GAS, P2X7 activation is not mediated by ATP but by a yet to be determined ligand for this receptor.

### P2X7-Dependent IL-1β Release Does Not Require P2X4 or the ADP-Ribosylating Protein CD38

**Figure 3:**
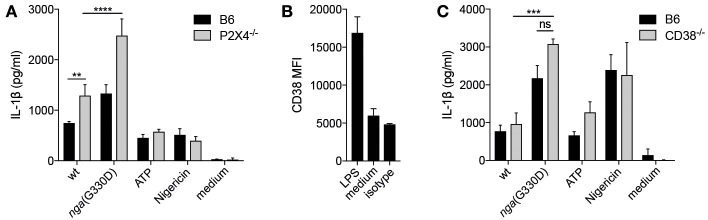
P2X7-dependent IL-1β release does not require P2X4 or the ADP-ribosylating protein CD38. **(A)** BMDMs of the indicated genotypes were LPS-primed and infected with wt or *nga*(G330D) GAS or treated with ATP or Nigericin. **(B)** CD38 expression on BMDMs after LPS-priming was analyzed by FACS. **(C)** BMDMs of the indicated genotypes were LPS-primed and infected with wt or *nga*(G330D) GAS or treated with ATP or Nigericin. Supernatants were analyzed for IL-1β by ELISA. ATP and Nigericin were used as controls for Nlrp3 inflammasome activation. Graphs show means ±SD for triplicate technical replicates and are representative of at least three independent experiments. medium: untreated cells. Significant differences are indicated by asterisks as follows: ^**^P < 0.01; ^***^P < 0.001; ^****^P < 0.0001. Values that are not significantly different are indicated (ns).

P2X7-dependent cytokine release can be potentiated by P2X4, another member of the P2X family (31). To investigate whether P2X4 was involved in the P2X7-dependent NADase-mediated effect on IL-1β release, BMDMs deficient for P2X4 were infected. IL-1β levels were significantly increased in infected P2X4^−/−^ compared to B6 BMDMs, but the absence of P2X4 did not reduce the amount of IL-1β released during *nga*(G330D) infections (Figure 3A), suggesting that the NADase-dependent changes to IL-1β secretion are independent of this receptor.

P2X7 function can also be altered by ADP-ribosylation of the receptor (32) and we found that the ADP-ribosyltransferase CD38 (33) was significantly upregulated during priming of B6 macrophages (Figure 3B). We thus hypothesized that CD38 may modulate P2X7 during *nga*(G330D) but not wt infection, as only wt bacteria would consume the substrate β-NAD^+^. However, we observed no difference in IL-1β release from infected CD38-deficient compared to B6 macrophages (Figure 3C).

### P2X7-Dependent IL-1β Secretion During NADase-Deficient Group A *Streptococcus* Infection Cannot be Explained by Altered Protein Degradation or Vesicular Release Patterns

**Figure 4:**
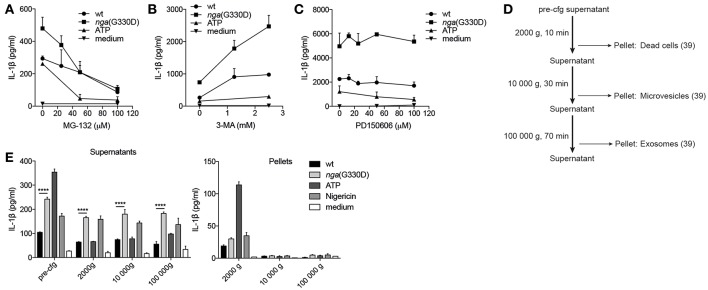
P2X7-dependent IL-1β secretion during NADase-deficient Group A *Streptococcus* infection cannot be explained by altered protein degradation or vesicular release patterns. B6 BMDMs were LPS-primed and infected with wt or *nga*(G330D) GAS, or left untreated (medium), in the presence of **(A)** MG-132, **(B)** 3-MA, or **(C)** PD150606. **(D)** Centrifugation setup schematic: supernatants were subjected to sequential centrifugation steps: 300 g, 5min (pre-cfg); 2000 g, 10 min; 10 000 g, 30 min and 100 000 g, 1 h. Pellets were harvested after the 2000 g, 10 000 g and 100 000 g steps. **(E)** Supernatants and pellets from LPS-primed, infected or untreated (medium) B6 BMDMs were isolated as in **(D)**. IL-1β levels were measured by ELISA. ATP and Nigericin were used as controls for Nlrp3 inflammasome activation. Graphs show means plus SD for **(E)** duplicate or **(A–C)** triplicate technical replicates and are representative of at least three independent experiments. Significant differences are indicated by asterisks as follows: ^****^P < 0.0001.

A possible explanation for the observed differences in IL-1β release is a selective or increased intracellular degradation of IL-1β during wt compared to *nga*(G330D) infection. The main degradative processes in cells are mediated by the proteasome or by autophagy, and previous studies have shown that IL-1β can be targeted for either pathway (34, 35). Thus, we added the proteasome inhibitor MG-132 or the broad autophagy inhibitor 3-MA to our infections and measured the effect on IL-1β release. To avoid interference with the priming step (36), the inhibitors were added to primed cells 30 min before infection with GAS. Interestingly, inhibition of proteasome activity (MG-132) resulted in a decrease in IL-1β release induced by both streptococcal strains, as well as by ATP, and at increased levels of inhibitor (50 μM), the difference in IL-1β release from wt and *nga*(G330D) infected cells was eradicated (Figure 4A). Together, our data suggest that IL-1β release in response to these stimuli, including release induced specifically by the NADase-deficient strain, may be dependent on degradation of one or several yet unknown proteins. In contrast, 3-MA significantly increased levels of released IL-1β upon infection with either strain (Figure 4B), suggesting, in line with previous studies, that IL-1β or one of the inflammasome components may be targeted for autophagosomal degradation during infection (35, 37). As levels of secreted IL-1β increased significantly from both wt- and *nga*(G330D)-infected BMDMs in the presence of 3-MA, we conclude that NADase-dependent inhibition of IL-1β release cannot be explained by selective autophagy-mediated degradation of IL-1β.

Many studies have suggested that P2X7 may be involved in regulating vesicle-mediated release of IL-1β (19) and a recent study propose that vesicular P2X7-dependent release pathways may involve calpains, a family of Ca^2+^-dependent proteases (38). However, in the presence of the selective calpain inhibitor PD150606 IL-1β release remained unchanged (Figure 4C), suggesting that secretion occurs independently of calpains.

To broadly investigate whether IL-1β may be released in vesicles during GAS infection, we subjected the supernatants of infected BMDMs to a series of centrifugation steps to collect vesicles by size (Figure 4D), lysed vesicle membranes using Triton-X and measured IL-1β. While our results indicate that a small portion of IL-1β can be found in dead cells (2000 g pellet), microvesicles (10,000 g pellet) and exosomes (100,000 g pellet) after infection (39), the vesicular release patterns induced by the two strains and the low total levels present in vesicles excludes differential release of IL-1β by vesicular transport (Figure 4E).

Together, these results suggest that the *nga*(G330D) strain does not inhibit protein degradation or induce a vesicle-mediated release pathway downstream of P2X7 that could explain the discrepancy between IL-1β levels induced by wt and *nga*(G330D) streptococci.

### P2X7-Mediated Release of IL-1β in Response to Group A *Streptococcus* Is Dependent on Permeabilization of the Plasma Membrane

**Figure 5:**
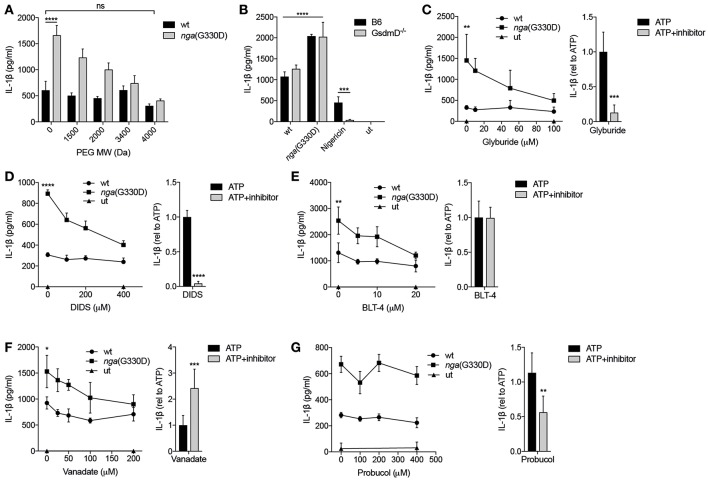
P2X7-mediated release of IL-1β in response to Group A *Streptococcus* is dependent on permeabilization of the plasma membrane. **(A)** LPS-primed B6 BMDMs were infected with wt or *nga*(G330D) GAS in the presence of poly-ethylene glycols (PEGs) of different molecular weight. **(B)** BMDMs of the indicated genotypes were LPS-primed and infected with wt or *nga*(G330D) GAS or treated with Nigericin. medium: untreated cells. **(C–G)** BMDMs were LPS-primed and infected with wt or *nga*(G330D) GAS (left panels) or stimulated with ATP (right panels) in the presence of **(C)** glyburide, **(D)** DIDS, **(E)** BLT-4, **(F)** Probucol, **(G)** Vanadate. ATP was used as a control for Nlrp3 inflammasome activation. medium: untreated cells. **(C–G)**, right panels Values are normalized to ATP-treated BMDMs. Supernatants were analyzed for IL-1β by ELISA. Graphs show means plus SD from three independent experiments **(C–G)**, right panels, or means plus SD for triplicate technical replicates representative of at least three independent experiments. Significant differences are indicated by asterisks as follows: ^*^P < 0.05; ^**^P < 0.01; ^***^P < 0.001; ^****^P < 0.0001. Values that are not significantly different are indicated (ns).

IL-1β has been suggested to be released through membrane pores or channels, or upon cell lysis (18). Polyethylene glycols (PEGs) can prevent cell lysis and death, if their diameter is larger than the that of pores in the cell membrane (40). In addition, PEGs may physically block passage of substances through membrane channels by steric obstruction (41). To investigate whether the *nga*(G330D) strain triggers a second release pathway in addition to the known SLO-dependent pathway that is activated by both strains, we employed a series of PEGs of increasing molecular weight, hypothesizing that these two pores should be blocked by PEGs of different size. In our experimental setup, BMDMs were infected with GAS for 90 min after which the bacterial suspension was removed and replaced with fresh media. Culture supernatants were then harvested and analyzed after 3 hrs. The presence of PEGs during either of the two phases of the experiment (90-min infection or 3 h post-infection incubation) had no effect on IL-1β release from cells infected with wt bacteria. In contrast, *nga*(G330D)-mediated release of IL-1β presented with a pronounced decrease correlating with increased PEG size, until IL-1β levels reached those of wt-infected cells (Figure 5A). This implies that the *nga*(G330D) strain induces the opening of an additional pore of smaller size than the SLO pore, and that this second passage is required for the release of the additional IL-1β. Of note, the effect of PEGs was similar regardless of whether they were present during the 90-min infection phase or the 3 h post-infection period (Supplementary Figure S2A). As only the P2X7-mediated, but not the SLO-mediated, IL-1β release seemed to be affected by PEGs, we expected IL-1β secretion from infected P2X7^−/−^ BMDMs to remain unchanged by PEGs during either phase of the experiment, which was indeed the case (Supplementary Figure S2A). When PEGs were present during the 90-min infection step, it had no effect on cellular integrity as measured by LDH release, while the addition of PEGs during the post-infection 3 h period clearly prevented leakage of cytosolic contents (Supplementary Figure S2B), suggesting that most cell death occurs during the later phase of the experiment and subsequent to IL-1β release. Consistent with this, the addition of the cytoprotectant glycine (42) efficiently blocked plasma membrane permeabilization during GAS infection but had no effect on the secretion of IL-1β (Supplementary Figure S2C). Thus, IL-1β release and cell death proceed with different kinetics and seem to be regulated by different pathways during GAS infection. To summarize, these data suggest that IL-1β secretion occurs through two different mechanisms during infection with *nga*(G330D) bacteria, one dependent of and one independent of P2X7, and that IL-1β and LDH are released through separate pathways during GAS infection.

Recent studies have highlighted the importance of the pore-forming protein Gasdermin D (GsdmD) in caspase-1-dependent IL-1β release and cell death (18). Interestingly, IL-1β release induced by streptococci was independent of GsdmD (Figure 5B). Cell death as measured by release of LDH was also largely independent on GsdmD (Supplementary Figure S2D), which is in agreement with previous studies suggesting that cell death during GAS infection occurs mainly through non-pyroptotic pathways (3, 5). Several other pore-forming proteins and transporters have been implicated in P2X7-dependent membrane permeabilization and/or IL-1β release (43, 44). Thus, we applied a number of inhibitors targeting membrane permeabilizers (Table 1) during GAS infection of macrophages and analyzed their effect on IL-1β release. Glyburide, an anti-diabetic drug used as an inhibitor of the ion and metabolite permeable channel pannexin-1 and the cholesterol efflux pump ABCA1—both membrane proteins that have been implicated in P2X7-mediated IL-1β release (43, 44)—dose-dependently reduced levels of IL-1β released from *nga*(G330D)-infected B6 macrophages, while it had no effect on IL-1β released from wt-infected B6 macrophages (Figure 5C). Similarly, the chloride transport inhibitor DIDS (Figure 5D), the lipid transport inhibitor BLT-4 (Figure 5E) and the ATPase inhibitor vanadate (Figure 5F), selectively affected release induced by the *nga*(G330D) strain (44–46). All four of these inhibitors have been shown to interfere with the ABCA1 channel, however, the antioxidant probucol—an inhibitor of ABCA1-mediated cholesterol efflux (47)—had no effect on IL-1β release in response to GAS (Figure 5G). Of note, when these inhibitors were applied to LPS-primed macrophages in which the inflammasome had been activated by ATP (Figures 5C–G, right panels), their effect was not always the same as on infected cells. Indeed, while all inhibitors apart from probucol dose-dependently decreased IL-1β release upon *nga*(G330D) infection, secretion upon ATP-driven inflammasome activation was reduced by glyburide, DIDS and probucol (Figures 5C,D,G; right panels), unaffected by BLT-4 (Figure 5E, right panel) and increased in the presence of vanadate (Figure 5F, right panel). These divergent effect patterns suggest that *nga*(G330D) infection drive IL-1β release through a different pathway as compared to ATP, which is also in line with our other findings presented here. Thus, while it is clear that *nga*(G330D) infection induces P2X7-mediated membrane permeabilization and that ABCA-1 is implicated by most of the used inhibitors, the identity of the membrane passage remains to be determined.

**Table 1:** Inhibitors used and their reported targets.

**Inhibitor**	**Targets**	**References**
Glyburide	Pannexin-1, ABCA1, P-gp, SR-BI, ATP-sensitive K^+^ channel, inflammasome activation	(43, 45, 48–50)
DIDS	Cl^−^ channels, ABCA1, inflammasome activation	(44, 48, 49)
BLT-4	SR-BI, ABCA1	(45)
Vanadate	ATPase activity (P-gp, ABCA1, ABCC1, ABCC2)	(46)
Probucol	ABCA1-mediated lipid efflux	(51)

## Discussion

The proinflammatory cytokine IL-1β is an important factor in mounting effective immune responses against many pathogens including GAS, illustrated e.g., by increased susceptibility to necrotizing GAS infections among patients receiving the IL-1R antagonist Anakinra (20) and of aggravated disease in GAS-infected IL-1 receptor (IL-1R)-deficient mice (21). However, the role of this cytokine in GAS infection is complex; in a forward genetics screen using a panel of intercrossed mice (C57BL/6xDBA/2J), increased susceptibility to GAS and enhanced bacterial growth correlated with increased IL-1β levels during infection (52). Moreover, type I interferon-deficient mice are unable to properly control IL-1β levels and thus rapidly succumb to GAS infection because of IL-1β-mediated pathology, even in the absence of bacterial expansion (21). Thus, to properly control infection and prevent detrimental tissue damage, it seems crucial that IL-1β is maintained at appropriate levels. The generation of bioactive IL-1β upon Nlrp3 inflammasome activation in response to GAS largely occurs due to the membrane-damaging toxin SLO (4, 5) We recently uncovered a role for the streptococcal β-NAD^+^-glycohydrolase NADase in limiting SLO-mediated IL-1β release (5), and propose that this represents a bacterial strategy to evade innate immune responses, which may at least in part explain the observation that increased expression of SLO and NADase correlates with increased bacterial virulence (51). Here we further explore the mechanistic basis for NADase-mediated modulation of IL-1β release and find that the expression of functional NADase allows the bacteria to avoid activation of the P2X7 receptor during infection. Indeed, a streptococcal strain expressing an enzymatically inactive version of NADase triggers P2X7-dependent membrane permeabilization leading to increased levels of IL-1β being released. Our results also align with a previous study suggesting caspase-1 activation induced by wt GAS is independent of the P2X7 receptor (4).

It is well-known from previous studies that ATP mediates P2X7-dependent activation of the inflammasome, but also has several other unrelated functions including the regulation of leaderless protein release (19). Interestingly, we observe P2X7-dependent increase of IL-1β levels but cannot detect any inflammasome activation *per se* downstream of P2X7, suggesting these processes may be mechanistically disconnected. The long cytoplasmic C-terminus of P2X7 harbors predicted binding sites for several interacting cytosolic components, also implying that this receptor is able to recruit distinct pathways upon activation, although it is unclear how these discrete downstream responses might be differentially triggered. It has been suggested that P2X7 when activated physically interacts with Nlrp3 leading to inflammasome complex assembly (53). Thus, the absence of P2X7-mediated inflammasome activation upon GAS infection could possibly be explained by SLO-mediated activation decreasing the amount of free Nlrp3 available for direct interaction with P2X7, i.e., substrate restriction may be one way the responses of this receptor can be regulated.

The unconventional protein secretion mediated by P2X7 has previously been coupled to several distinct mechanisms, with one release concept relating to the permeabilization of and subsequent transport across the plasma membrane (54). How this P2X7-mediated membrane permeabilization occur is debated (26); one hypothesis states that the P2X7 receptor itself forms a large pore permeable to molecules up to 900 Da (55), and the other that macropore formation is mediated by the recruitment of one or several accessory proteins (26). Direct release of IL-1β through the P2X7 receptor complex is unlikely as the diameter of the mature cytokine is significantly larger than the estimated P2X7 pore size [4.5 vs. 1.4 nm (56, 57)], suggesting the involvement of accessory proteins. Two lines of data in our study suggest that IL-1β release during NADase-deficient GAS infection in fact occur through more than one mechanism: (1) absence of P2X7 does not completely abrogate IL-1β release during *nga*(G330D) infection, but instead decreases levels to those observed during wt infection, and (2) the excess IL-1β that is released during *nga*(G330D) infection can be blocked by PEGs while the wt-induced release cannot. The study of IL-1β release has revealed multiple pathways seemingly dependent on e.g., cell type and inducing stimulus and it has been suggested that this versatility may represent biological diversity and/or possibly reflect cell health (54). Our data also highlight another level of complexity as they indicate that multiple mechanisms at once may govern IL-1β release from a single cell, which seems a likely representation of what might occur during infection *in vivo* considering the intricacy of host-pathogen interactions and the promiscuity of IL-1β release pathways. Our studies also emphasize the importance of complementing the use of more reductionist systems with infection models for our appreciation of how eukaryotic cells sense, integrate and decode the multitude of signals occurring simultaneously during infection.

The P2X7-dependent IL-1β release observed during *nga*(G330D) infection is sensitive to several of the ion and ABC channel inhibitors tested. Although our data does not allow us to identify the components involved, or propose whether inhibition relates to the actual release of IL-1β or an upstream regulatory process, it clearly indicates a role for an accessory protein during IL-1β release in response to GAS. Of note, some of these inhibitors have also been reported to directly target inflammasome activation (48). However, as we observe an exclusive effect on *nga*(G330D)-induced release (wt-induced release remains unchanged), and as we cannot detect any differences in inflammasome activation between wt and *nga*(G330D) infection, we conclude that the inhibitors in our infections rather cause a disturbance of the secretion process.

Until recently, cytokine secretion and pyroptotic cell death were generally considered linked and default processes downstream of inflammasome activation. It has also been suggested that IL-1β release is a mere consequence of cell lysis (58). In line with more recent studies challenging these views, we observed differential PEG-induced inhibition patterns for IL-1β secretion and cell death (as measured by LDH release). Indeed, while IL-1β secretion is equally affected regardless of whether PEGs are present during or after the infection, cell death is only inhibited when PEGs are present during the post-infection phase. Moreover, IL-1β secretion upon *nga*(G330D) or wt infection is differentially affected by PEGs, whereas the pattern of cell death inhibition seems independent of infecting strain. These observations let us conclude that IL-1β release, but not cell death, is regulated by streptococcal NADase and P2X7; and that IL-1β secretion and cell death are kinetically separate events during GAS infection of macrophages. Further, they allow us to hypothesize that PEGs may block IL-1β secretion by steric hindrance, as inhibition occur also when PEGs are present only during infection and removed for the post-infection phase, but that this does not seem to be the case for cell death.

In summary, our results indicate that macrophages can release IL-1β through several pathways in response to GAS, and that one of these pathways is P2X7-dependent and inhibited in the presence of a functional NADase enzyme. This pathway involves membrane permeabilization induced by the P2X7 receptor but seems to be disconnected from inflammasome activation *per se*. Intriguingly, our data suggest that GAS has evolved strategies to specifically evade activation of the P2X7 receptor, making it of general interest to further investigate the role of the P2X7 receptor in GAS pathogenesis.

## Materials and Methods

### Bacterial Strains and Growth Conditions

GAS strain 854 is an M1 strain isolated from a patient with a retroperitoneal abscess (59). The isogenic mutant strains used in this study have been described previously (7), and was a generous gift from Michael R Wessels. The Δ*slo* strain harbors a deletion of the *slo* gene and *nga*(G330D) expresses an enzymatically inactive variant of the NADase toxin. GAS strains were grown in Todd-Hewitt broth supplemented with 0.5% yeast extract (THY) at 37°C, 5% CO_2_. For infection, overnight THY cultures were re-inoculated in fresh THY and grown to late exponential phase (OD_600_ = 1.1–1.3), collected by centrifugation and washed with phosphate-buffered saline (PBS) before diluting to the desired multiplicity of infection (MOI).

### Mice

All genetically modified mouse strains [P2X7^−/−^ (60), P2X4^−/−^ (61), GsdmD^−/−^ (62), and CD38^−/−^ (63)] were on a C57BL/6 (B6) background. B6 mice were purchased from the animal facility of the Biomedical Center, Lund University. Genetically modified mice or bone marrow from modified mice were kindly provided by Frances E Lund (CD38^−/−^), GlaxoSmithKline (P2X4^−/−^), and Russell E. Vance (GsdmD^−/−^). P2X7^−/−^ mice were purchased from Jackson Labs (originally made by Pfizer). All experiments were conducted in accordance to the Malmö/Lund animal ethics committee.

### Generation and Infection of Bone Marrow-Derived Macrophages

Bone marrow was isolated from murine femurs and tibiae and progenitor cells were differentiated into bone marrow-derived macrophages (BMDMs) for 7 days in RPMI 1640 (Gibco) supplemented with 10% FBS (Sigma), 2.5 mM L-glutamine (Gibco) and macrophage colony-stimulating factor at 37°C in 5% CO_2_. For infection experiments, BMDMs of indicated genotypes were primed with 0.1 μg/ml LPS (Sigma) for 15 h and infected at MOI 1:30 before centrifugation at 300 g for 5 min and 1.5 h incubation at 37°C in 5% CO_2_. At this point the bacterial suspension was replaced with fresh media containing 300 μg/ml gentamicin (Sigma) to kill extracellular bacteria and the BMDMs were incubated for another 3 h. As positive controls of inflammasome activation, LPS-primed uninfected macrophages were treated with ATP or Nigericin (Sigma) the last 30 min before experimental end point. Supernatants were harvested after centrifugation at 300 g for 5 min to clear debris. Where indicated, BMDMs were infected in the presence of various compounds. A-740003 (Tocris), Glyburide (Sigma), 4,4^−/−^-diisothiocyanatostilbene-2,2^−/−^-disulfonic acid disodium salt hydrate (DIDS) (Sigma), Sodium metavanadate (Sigma), BLT-4 (Sigma), Probucol (Sigma) and PD-150606 (Tocris) were added 30 min before infection and were also present during the 90 min infection. Nicotinamide (NAM) (Sigma), ADP-ribose (ADPR) (Sigma), NAD^+^ (Sigma), 3-MA (Sigma), Glycine (Sigma) and MG-132 (Merck) were present during the 90 min infection. PEGs (Sigma, ICN Biomedicals, Merck) were present during the 90 min infection or the 3 h incubation. Apyrase (Sigma) was present during the whole infection (90 min+3 h).

### Differentiation and Infection of THP-1 Cells

THP-1 cells were cultured in RPMI 1640 supplemented with 10% FBS, 2 mM L-glutamine, 1 mM sodium pyruvate, 5 mM HEPES, 4.5 mg/ml D-glucose and 0.05 mM 2-mercaptoethanol at 37°C in 5% CO_2_. Differentiation into macrophages was acquired by stimulation with 5 ng/ml phorbol 12-myristate 13-acetate (PMA) for 48 h. GAS infection of THP-1 cells was performed as described above following 24 h priming with 5 ng/ml LPS. For inhibition of the P2X7 receptor, A-740003 (Tocris) was added 30 min before infection and was present throughout the experiment.

### Cytokine Analysis

Supernatants from infected BMDMs or THP-1 cells were analyzed for IL-1β or IL-1α using ELISA kits (BD Biosciences and R&D Systems) or cytometric bead array (BD Biosciences), respectively, according to the manufacturer's instructions. Of note, we commonly detect fluctuations in absolute IL-1β levels between experiments, likely due to features of the ELISA kits. Importantly, the ratio of IL-1β induced by different treatments remains stable throughout all experiments performed.

### Caspase-1 Activity Assay

Cleared supernatants were analyzed using Caspase-Glo Inflammasome Assay (Promega), according to the manufacturer's instructions. Luminescence was measured on a Varioskan LUX plate reader.

### Flow Cytometry

For surface staining, cells were infected or treated as indicated and washed with PBS, incubated in blocking buffer (10% rabbit IgG or FBS in PBS) and stained with relevant antibodies. For analysis of intracellular and cell-associated bacteria, GAS cultures were washed, and the bacteria labeled with an Alexa Fluor 660 NHS Ester (Life technologies). The following antibodies and isotype controls were used: anti-mouse CD38-FITC (BD Biosciences, cat #558813, clone 90), rat IgG2a isotype control-FITC (eBioscience, cat #11-4321-82, clone eBR2a), anti-Streptococcus A-FITC (LSBio, cat #LS-C86701), anti-P2X7 receptor (extracellular) (Alomone, cat #APR-008), purified rabbit IgG isotype control (Life technologies, cat #026102). A Zenon kit (Life technologies) was used to label the P2X7 antibody and the purified rabbit IgG with the fluorescent conjugate Alexa Fluor 647. Stained cells were washed and fixated with 4% paraformaldehyde (PFA), acquired on a BD LSR II or Accuri C6 flow cytometer and analyzed using FlowJo.

### Cytotoxicity Assay

Release of the cytosolic protein lactate dehydrogenase (LDH) was used as a mean to assess cell death. Cleared supernatants were analyzed using the CytoTox 96 assay (Promega) according to the manufacturer's instructions. Untreated cells were used to determine background LDH levels, and lysed, untreated cells were used as a reference for maximal LDH release.

### ATP Measurement

Cleared supernatants or whole well contents from infected BMDMs were analyzed using CellTiter-Glo Luminescent Cell Viability Assay (Promega), according to the manufacturer's instructions. Luminescence was measured on a Varioskan LUX plate reader.

### Isolation of Microvesicles and Exosomes

BMDMs were seeded at 1 ×10^6^ cells/well in 6-well plates and infected as above. After infection, supernatants were subjected to the following centrifugation steps to clear supernatant of debris and isolate secreted vesicles: 300 g, 5 min at RT; 2,000 g, 10 min at 4°C; 10 000 g, 30 min at 4°C and 100 000 g, 70 min at 4°C. The ultracentrifugation was performed with a swinging bucket rotor in an L-80 Ultracentrifuge (Beckman Coulter). Pellets collected from the last three centrifugation steps were resuspended in PBS. Supernatants and pellets were treated with 1% Triton-X (Sigma) for 30 min on ice prior to cytokine analysis.

### Data Processing and Statistical Analysis

Statistical calculations were performed using one or two-way ANOVA. *P*-values are indicated by asterisks: ^*^p ≤ 0.05; ^**^p ≤ 0.01; ^***^p ≤ 0.001; ^****^p ≤ 0.0001.

## Data Availability

All datasets generated for this study are included in the manuscript and/or the Supplementary Files.

## Ethics Statement

This study was carried out in accordance with the recommendations of Lund/Malmö Animal Ethics Committee. The protocol was approved by the Lund/Malmö Animal Ethics Committee.

## Author Contributions

EW and JP conceived and designed the experiments and analyzed the data. EW and CV performed the experiments. DY generated and contributed essential materials. EW and JP wrote the paper. All authors critically reviewed the study.

### Conflict of Interest Statement

The authors declare that the research was conducted in the absence of any commercial or financial relationships that could be construed as a potential conflict of interest.
