# Supplementary material for: The Secreted Virulence Factor NADase of Group A Streptococcus Inhibits P2X7 Receptor-Mediated Release of IL-1β
Source: Front Immunol. 2019 Jun 18;10:1385. doi: 10.3389/fimmu.2019.01385 (PMC6591467; doi:10.3389/fimmu.2019.01385)
Supplement: Supplementary file 1 [file Image_1.pdf]

Supplementary Figure S1

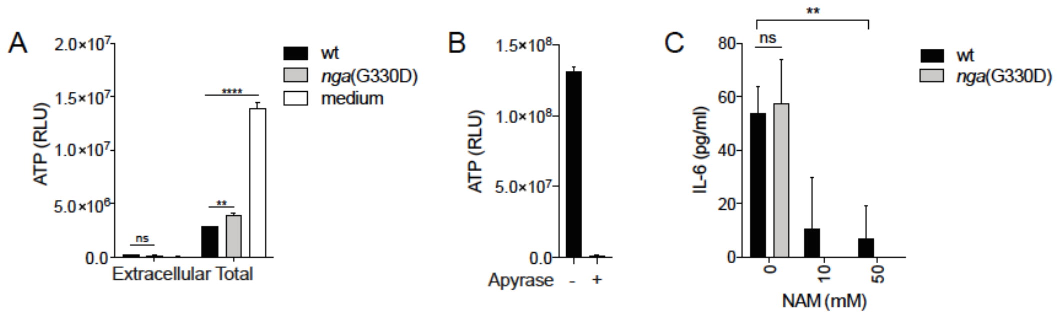

Supplementary Figure S1. P2X7-dependent IL-1β release occurs independently of extracellular ATP or β-NAD$^+$ cleavage products. (A) LPS-primed or untreated P2X7$^{-/-}$ BMDMs were infected with wt or *nga*(G330D) GAS and ATP content analyzed by a luminescence-based assay. **(B)** 5mM ATP was treated with Apyrase for 30 minutes and ATP content was analyzed by a luminescence-based assay. **(C)** LPS-primed BMDMs were infected with wt or *nga*(G330D) GAS in the presence of NAM. Supernatants were analyzed for IL-6 by ELISA. Graphs show means plus SD for triplicate samples and are representative of at least two experiments. Medium: unprimed, uninfected BMDMs. Significant differences are indicated by asterisks as follows: **, P < 0.01; ****, P < 0.0001. Values that are not significantly different are indicated (ns).

Supplementary Figure S2

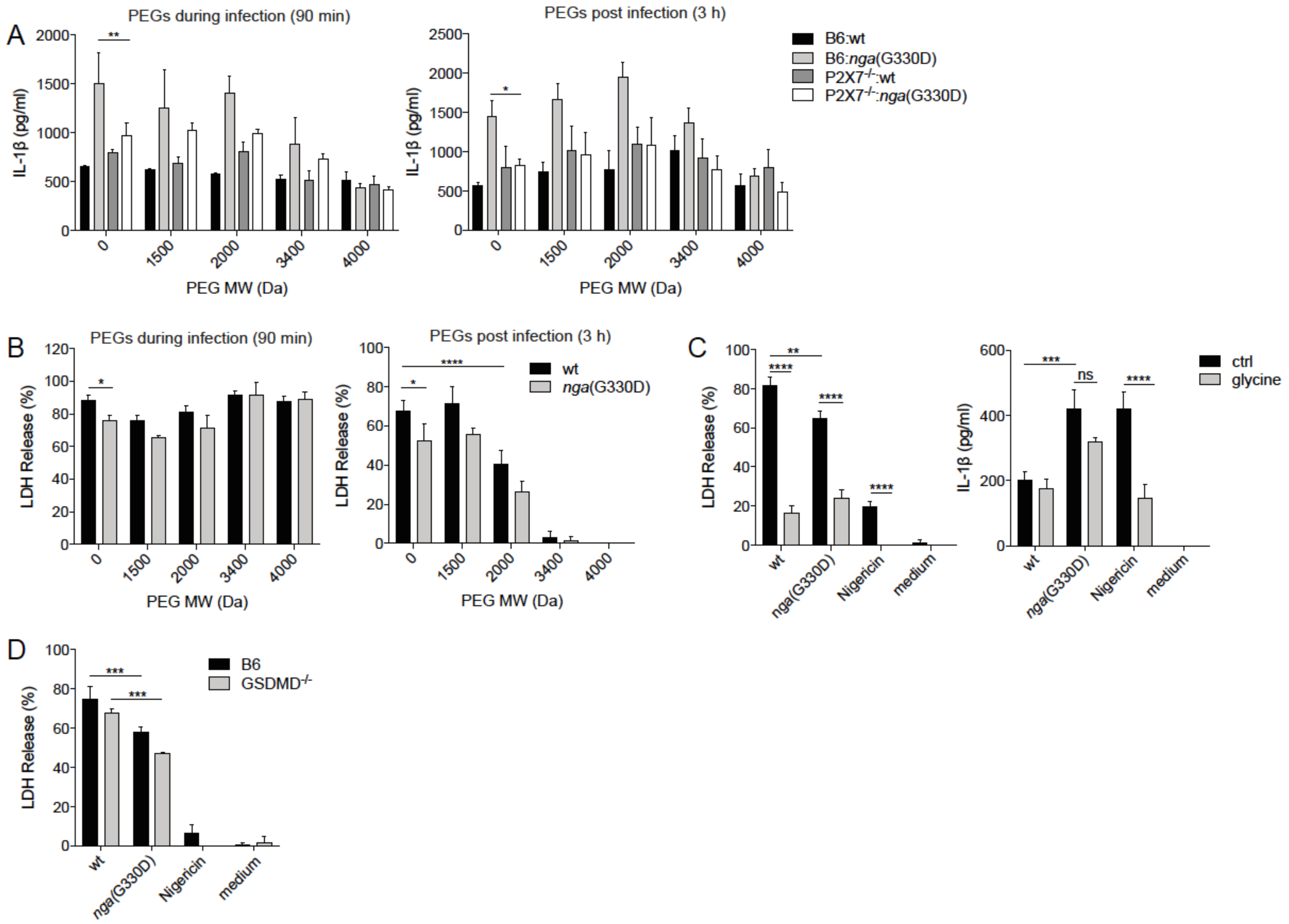

Supplementary Figure S2. P2X7-mediated release of IL-1β in response to Group A *Streptococcus* occurs independently of cell death. (A) LPS-primed BMDMs of the indicated genotypes were infected with wt or *nga*(G330D) GAS in the presence of PEGs of different molecular weight during the time periods indicated. Supernatants were analyzed for IL-1β by ELISA. **(B)** LPS-primed BMDMs were infected with wt or *nga*(G330D) GAS in the presence of PEGs of different molecular weight during the time periods indicated. Cell death was analyzed by measuring LDH release. **(C)** LPS-primed or untreated BMDMs were infected with wt or *nga*(G330D) GAS in the presence of glycine. Cell death (left panel) was analyzed by measuring LDH release. IL-1β release (right panel) was analyzed by ELISA. **(D)** LPS-primed or untreated BMDMs of the indicated genotypes were infected with wt or *nga*(G330D) GAS. Cell death was analyzed by measuring LDH release. Graphs show means plus SD for **(A)** duplicate or **(B-D)** triplicate samples and are representative of at least two experiments. Nigericin was used as a control for inflammasome activation and glycine-sensitive cell death. Medium: unprimed, uninfected BMDMs. Significant differences are indicated by asterisks as follows: *, P < 0.05; **, P < 0.01; ***, P < 0.001; ****, P < 0.0001. Values that are not significantly different are indicated (ns).
